# Meeting Rural Demand: A Case for Combining Community-Based Distribution and Social Marketing of Injectable Contraceptives in Tigray, Ethiopia

**DOI:** 10.1371/journal.pone.0068794

**Published:** 2013-07-12

**Authors:** Ndola Prata, Karen Weidert, Ashley Fraser, Amanuel Gessessew

**Affiliations:** 1 Bixby Center for Population, Health and Sustainability, School of Public Health, University of California at Berkeley, Berkeley, California, United States of America; 2 Department of Gynaecology and Obstetrics, Mekelle University College of Health Sciences, Mekelle, Ethiopia; Indiana University, United States of America

## Abstract

**Background:**

In Sub-Saharan Africa, policy changes have begun to pave the way for community distribution of injectable contraceptives but sustaining such efforts remains challenging. Combining social marketing with community-based distribution provides an opportunity to recover some program costs and compensate workers with proceeds from contraceptive sales. This paper proposes a model for increasing access to injectable contraceptives in rural settings by using community-based distributers as social marketing agents and incorporating financing systems to improve sustainability.

**Methods:**

This intervention was implemented in three districts of the Central Zone of Tigray, Ethiopia and program data has been collected from November 2011 through October 2012. A total of 137 Community Based Reproductive Health Agents (CBRHAs) were trained to provide injectable contraceptives and were provided with a loan of 25 injectable contraceptives from a drug revolving fund, created with project funds. The price of a single dose credited to a CBRHA was 3 birr ($0.17) and they provide injections to women for 5 birr ($0.29), determined with willingness-to-pay data. Social marketing was used to create awareness and generate demand. Both quantitative and qualitative methods were used to examine important feasibility aspects of the intervention.

**Results:**

Forty-four percent of CBRHAs were providing family planning methods at the time of the training and 96% believed providing injectable contraceptives would improve their services. By October 2012, 137 CBRHAs had successfully completed training and provided 2541 injections. Of total injections, 47% were provided to new users of injectable contraceptives. Approximately 31% of injections were given for free to the poorest women, including adolescents.

**Conclusions:**

Insights gained from the first year of implementation of the model provide a framework for further expansion in Tigray, Ethiopia. Our experience highlights how program planners can tailor interventions to match family planning preferences and create more sustainable contraceptive service provision with greater impact.

## Introduction

Addressing family planning needs has important human rights implications and remains an essential part of “the unfinished agenda” for achieving global development goals [1]–[4]. In the developing world, approximately 222 million women would like to delay or stop childbearing but are not using any method currently and, thus, have an unmet need for family planning [5]. Several important barriers to contraceptive use have been identified including demand, knowledge or awareness, access or supply, and method mix, all of which are greater for rural and poor populations [6]–[8]. In Sub-Saharan Africa, restricting injectable contraceptives to clinic-based provision has limited access to family planning services. Policy changes have begun to pave the way for distribution of injectable contraceptives outside facilities but sustaining such efforts remains challenging [9], [10].

This paper proposes a model for increasing rural access to injectable contraceptives by using community-based distributers as social marketing agents and incorporating financing systems to improve sustainability of community-based distribution. We have completed one year of applying this model in rural Ethiopia by adapting and scaling up a pilot project, which tested the community-based distribution of injectable contraceptives in the same setting [11]. Drawing on our initial field experiences, we report preliminary findings to illustrate the opportunities and challenges involved in scaling up community-based distribution of injectable contraceptives paired with social marketing strategies.

## Background

Injectable contraceptives are the most popular contraceptive method in Sub-Saharan Africa, with an estimated 9 million users contributing 43% of total method mix in the region [12]. Injectable contraceptives are among the most effective contraceptive methods, after intrauterine devices, implants, and sterilization [9]. A single dose of the injectable contraceptive depot medroxyprogesterone acetate (DMPA) offers 3-month protection and is female-controlled, non-coital dependent, and reversible. Equally important is the potential for inconspicuous use of DMPA, which may be critical for women, particularly those in rural communities interested in covert family planning [13], [14].

Community-based distribution of contraceptives has been an invaluable strategy for extending family planning services, successfully increasing contraceptive prevalence, and decreasing discontinuation in developing countries since the 1950s [8], [9], [15], [16]. This service delivery approach remains indispensable for addressing the tremendous unmet need among rural poor women [10], [17], [18]. Engaging community health workers for delivering health services in rural settings can be very cost effective [19], [20]. Most family planning programs use trained volunteer community health workers who are recognized and respected within rural communities to provide contraceptive outreach, screen and counsel prospective clients, and dispense methods [10]. Such programs can simultaneously include information, education, and communication (IEC) campaigns, which increase contraceptive knowledge, allay fears, and often generate new demand [9]. Community-based distribution reduces barriers to access while also reducing demands on the time of existing, more highly trained providers, and costs to facilities [8]–[10].

Asian and Latin American community-based distribution programs have provided DMPA at the community level since the 1970s [16], [21], [22], but in Africa, restrictive laws have limited DMPA provision to clinics until very recently [10], [23], [24]. Studies from Uganda, Madagascar, Malawi and Ethiopia have demonstrated that with sufficient training, community health workers can provide DMPA injections with comparable safety, acceptability, and continuation rates as clinic based providers [11], [14], [16], [25]–[27]. Furthermore, a WHO technical consultation concluded that the evidence base supports the scale-up of community-based provision of injectable contraceptives [9].

In developing countries, while government programs remain the largest family planning providers, the private sector is increasingly meeting the needs of poor populations [28], [29]. Social marketing, or the sale of subsidized, branded contraceptives at widespread private sector outlets, is a practical family planning service delivery approach, which can be a powerful tool for increasing knowledge and access. Using commercial marketing techniques, social marketing makes the product available and affordable while linking it to a communications campaign geared to effect sustainable behavioral change [29]–[31]. Therefore, different than public sector provision of contraceptives, social marketing of contraceptives can shift toward demand-driven approaches rather than supply-driven approaches. In 2011, contraceptive social marketing programs were operating in 69 developing countries, supplying 53 million couple-years of protection, including 6 million couple-years of protection from injectable contraceptives [32]. Over the past decade, social marketing has accounted for an increasing share of the contraceptive method mix. These trends have been linked to income generation, which fosters program stabilization [29], [33].

Historically, social marketing has been implemented in urban settings where exposure to the mass media is high, private shops and pharmacies are well-established and prevalent, and demand for contraception is strong [34]. Yet, in many developing countries, the majority of the population still lives in rural areas with limited access to media and distribution networks. Rural populations represent an enormous and potentially untapped market for socially marketed contraceptives with substantial existing unmet demand and considerable potential for demand creation [35].

### Rationale for Combining Community-based Distribution with Social Marketing

Community-based distribution has the potential to alleviate strains on overburdened clinic staff and extend family planning access to rural areas but volunteer worker retention and financial concerns threaten long term program sustainability [10], [36]. Numerous experts have recommended compensation to motivate community-based distribution workers [10], [37], [38]. Unfortunately, national governments cannot afford to remunerate large numbers of community-based distribution workers. Funding for family planning interventions is under constant threat and donor support alone has been insufficient to satisfy increasing demand [39], [40]. Innovative service delivery approaches and strategies are needed to address these challenges.

Social marketing has the potential to increase sustainability by enabling financing, cost sharing, and offsetting costs, but distribution networks have typically been limited to commercial outlets concentrated in urban areas. By engaging community-based distribution workers, social marketing programs would tap into existing community networks [30], [41]. Combining social marketing with community-based distribution offers an opportunity to recover some program costs and compensate workers with some of the proceeds from the contraceptive sales. Subsidies would still be necessary, but the costs would be reduced by the volume of sales.

## Methods

### Ethics Statement

Human subjects approval for baseline data collection was provided by the Committee for Protection of Human Subjects (CPHS) at the University of California Berkeley (CPHS Protocol ID 2011/07/3465). We obtained verbal consent from all women participating in the baseline data collection. The greatest risk to the subjects in our study was a breach of confidentiality and the signed written consent form would be the only record linking the subject to the research. The majority of subjects were illiterate, and therefore, the verbal consent form, approved by CPHS, was read to the woman with ample opportunity for questions and concerns. If she verbally consented to participate, the interviewer marked a box indicating verbal consent was obtained and then signed and dated the form, which was attached to each completed survey.

In Ethiopia, girls under 18 years old are legally endowed to seek reproductive health services without parental consent, including family planning and abortion services. Therefore, imposing a parental consent in this study would place an undue burden upon youth who are seeking care afforded to them under Ethiopian law. In recruiting girls 15–17 years old for the survey, the interviewer used a specially designed recruitment script to ask the head-of-household, if present, if she could speak with the minor about participating in the survey. Verbal assent (rather than written consent) was required and documented in the same manner and for the same reasons as described above for women 18 years and older. The CPHS at the University of California, Berkeley approved this consent procedure, including the parental consent waiver and the recruitment scripts.

**Figure 1:**
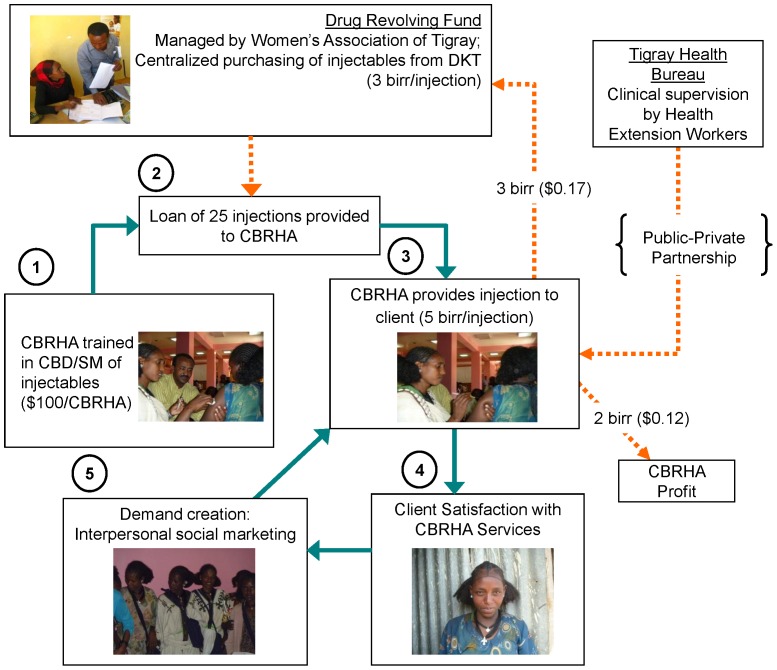
Concept design for linking community-based distribution and social marketing in Tigray, Ethiopia.

The Tigray Regional Health Bureau does not have a formal ethics committee and supported approval through CPHS at University of California Berkeley. All photographs in the model featured in Figure 1 depict project team members such as CBRHAs, including the “Client satisfaction with CBRHA services” picture of a CBRHA/client, who provided written informed consent (as outlined in the PLoS consent form) specifically for her image to be published in her dual role.

### Study Setting

Situated in the northernmost region of Ethiopia, Tigray is home to over four million people, and typical of much of country, 81% of the residents live in rural areas [42]. In 2011, the total fertility rate in Tigray was 4.6 [43]. Modern contraceptive prevalence in Tigray was 21% and injectable contraceptive prevalence was 13% among married women, representing more than three fifths of the modern method mix [43]. Yet unmet need for family planning in the region was 22% and less than half of the total demand for family planning was satisfied [43]. Efforts to increase access to family planning services in rural areas began in the early 1990s in Ethiopia. Community-based reproductive health agents (CBRHAs) are volunteer lay health workers who have been mobilized in the past to provide basic family planning education, services and referrals in rural Ethiopia. However, early community-based distribution efforts were hampered by misperceptions of religious prohibition against family planning, lack of awareness, and worker supervision and compensation issues [44]. More recent community-based distribution projects have addressed some of these challenges [11], [36], [45]. A pilot study conducted by Prata et al. recommended that CBRHAs should be authorized to administer DMPA [11]. Unfortunately, long-term sustainability remains a pressing, yet unresolved issue [45]. Contemporary program experience suggests that including injectable contraceptives may be essential for community-based distribution program viability [46]. Contraceptive social marketing has been used in Ethiopia since 1990 with injectable contraceptives representing the second largest share of social marketing sales in 2010 [32].

This intervention was implemented in three woredas (districts) of the Central Zone of Tigray, Ethiopia and program data has been collected from November 2011 through October 2012.

### Concept Design

Displayed in Figure 1 is the model of our concept design, which combines social marketing strategies with community-based distribution of DMPA. This model of community-based distribution of DMPA addresses financial sustainability through the application of social marketing and a drug revolving fund, which with seed money can effectively be used to leverage additional resources for a greater impact on contraceptive prevalence. Our methodology builds upon the aforementioned pilot study in Ethiopia, which demonstrated the safety, acceptability, and effectiveness of community-based distribution of DMPA [11], while also incorporating a fee-for-service strategy similar to the approach used in Madagascar where community-based distribution workers charged a small fee for their services to recover some program costs and provide compensation to the distributors [27]. Implementing agencies include the Bixby Center at University of California Berkeley, Mekelle University, and the Women’s Association of Tigray, in collaboration with the Tigray Regional Health Bureau.

As illustrated in Figure 1, the first step of the model is training of CBRHAs in community-based distribution and social marketing of injectable contraceptives. A drug revolving fund, initially created with project funds ($10,500 USD), is managed by the Women’s Association of Tigray and replenished with the money paid back into the system by CBRHAs providing injections for a fee. The money invested in the drug-revolving fund is used by the Women’s Association of Tigray for centralized purchasing of additional DMPA from DKT International-Ethiopia (DKT-Ethiopia or DKT). At the beginning of the training, CBRHAs are provided with a micro-loan of 25 injections of DMPA from the drug revolving fund. The price of a single dose of DMPA credited to a CBRHA is 3 birr ($0.17), which is the cost of one dose of DMPA purchased from DKT-Ethiopia. CBRHAs provide DMPA to women for 5 birr ($0.29). This amount was determined with willingness-to-pay data from the pilot study in Tigray, Ethiopia [11]. Of the 5 birr payment received by the CBRHA for each injection administered, 3 birr is returned to the drug revolving fund, with 2 birr ($0.12) profit retained by the CBRHA.

Payment exceptions could be made by CBRHAs on a case-by-case basis for certain circumstances as outlined during training. For example, if a woman was too poor and unable to reach the health post (for free services); or was not in a position to ask her husband or family for money; or in case of an adolescent, needed private services fearing stigma; or for any other reasons considered necessary by CBRHAs. CBRHAs documented the reasons for each case as instructed during training and were not compensated for free injections.

The injectable contraceptive product used in this project is distributed by DKT-Ethiopia and is marketed under the brand name *Confidence*. It is available throughout the country and is distributed by DKT’s extensive commercial network. DKT’s distribution is leveraged to allow CBRHAs to sell injectable contraceptives with cost reductions through bulk purchases by the Women’s Association of Tigray.

Social marketing campaigns by DKT-Ethiopia, which are active in urban areas, were expanded to attract rural women to the branded injectable contraceptive, *Confidence*. Social marketing is used to create awareness and generate demand. However, the social marketing associated with this project focuses on promoting CBRHAs as a valued service delivery option, with less emphasis on brand recognition of the DMPA commodity. This is a natural adaptation of the classic contraceptive social marketing model to the rural setting, since CBRHAs are respected and valued community leaders and confidentiality of services is paramount for many rural women. Notably, the pilot study in Ethiopia revealed women’s preference for receiving contraceptive services in their home and the role of CBRHAs in increasing rural access [11]. Demand is generated by client satisfaction and interpersonal communication and marketing within the communities. Satisfied clients share their experiences with other women in the community.

### Implementation of Model

#### Training

Officials from the Women’s Association of Tigray identified CBRHAs from three woredas of the Central Zone in Tigray and invited them to participate in training workshops on the provision and social marketing of DMPA, which were conducted by clinical staff and experts from Mekelle University, the Tigray Regional Health Bureau, and University of California, Berkeley. Each training session lasted three days, including clinical and logistical components. CBRHAs that successfully completed the classroom training progressed to the clinic-based practical training, which emphasized safe injection technique. CBRHAs were trained on counseling related to all family planning methods; screening procedures for DMPA; procedures for reporting side effects adverse events and other morbidities. CBRHAs were also trained in the management of funds, and DMPA resupply. CBRHAs were provided information on how to refer clients to the nearest health facility if they were ineligible for DMPA or for long-acting methods. The project emphasized reaching new family planning adopters, regardless of the chosen method, and no DMPA recruitment targets were established for CBRHAs. A one-day refresher training was held approximately nine months after the first training. The training activities cost approximately $120 per CBRHA, excluding curriculum development. Upon completion of the training, participating CBRHAs were authorized by the Tigray Health Bureau to administer injectable contraceptives. CBRHAs, who are already linked to the health posts in their villages, were instructed to report all clinical issues which clients reported experiencing to their assigned local health posts where public sector health workers provided clinical supervision.

#### Data collection

Quantitative methods were used to examine important feasibility aspects of the intervention. Prior to implementation of the intervention, community-level baseline data was collected. The structured questionnaire used in baseline data collection was largely based on the demographic, fertility, and family planning sections of the Demographic and Health Survey (DHS) with additional questions added to gain more information on women’s access to and willingness to pay for DMPA. The instrument, developed in English, was thoroughly discussed with research partners in Ethiopia. After a final draft of the questionnaire was agreed upon, it was translated into Tigrinya and back-translated into English. A local team of 18 current students and recent graduates in public health at Mekelle University were recruited to be interviewers and 3 were selected to be supervisors. They all participated in a three day interviewer training that included a range of sessions from human subjects training to step-by-step discussion of each survey question. The interviewers piloted the instrument and received feedback from trainers on adherence to protocol. No issues requiring additional changes to the instrument were necessary after pilot testing.

The sampling design for baseline data collection was intended to provide data that was representative of the Central Zone of Tigray, Ethiopia. A multi-stage cluster random sampling was employed. With probability proportional to size, a total of three woredas (districts) in the zone were randomly selected to be part of the sample for the Central Zone. All women 15–49 years of age in the randomly selected households were eligible to participate in the study. Interviewers and supervisors were sent to the three selected woredas in teams of five to six interviewers and one supervisor. Women who consented to participate were interviewed in Tigrinya in a private location of their own choosing in their homes or nearby. The average interview took 45–60 minutes and was conducted with complete confidentiality. Data collection took a total of 15 days and the final sample consisted of 1490 women. Our response rate was 99%, with all respondents providing verbal informed consent, and the remaining 1% refusing to be interviewed. Data from the 1490 surveys was entered into a database using Epi Info Version 3.5.3 and coded at the University of California Berkeley. A native Tigrinya speaker in Berkeley assisted in the translation of ‘other’ responses. Descriptive statistics were used to describe the target population and their knowledge of and access to modern contraceptives, fertility and contraceptive method preference, as well as provider preference for receiving family planning services. The survey also included questions regarding knowledge, attitudes, and practices related to DMPA. Women with an unmet need for contraception were those who were married, fecund, did not want a/another child or did not want one for at least two years, but were not using contraception, per the DHS definition. Additionally, women whose current or last pregnancy was mistimed or unwanted were also categorized as having an unmet need for the purpose of this analysis. The final report for the baseline survey, which provides detailed information on sampling design and data collection, is available on the Bixby Center website [47] and the data are available upon request.

A written, anonymous Provider Characteristics Survey was completed by CBRHAs participating in the training to gather demographic data and information related to their previous experience with the provision of family planning services. For example, providers were asked if they ever received family planning training and if they thought this training would improve their services in the community. They were also asked if they were comfortable providing injections to unmarried women or adolescents. The structured survey, which included 15 close-ended questions, was ultimately used to establish the profile of CBRHAs involved in the project. The Provider Characteristics Survey was completed in private by each CBRHA on the last day of the training, unless she needed assistance because of her literacy level, with a response rate of 99%. While one CBRHA’s survey was unaccounted for (1% missing), no provider declined to complete the survey. Only 9% of CBRHAs reported having no education and they received the assistance of their choice to complete the survey.

#### Supervision

Direct supervision of CBRHAs was conducted by Health Extension Workers, government employees who oversee health post activities. Health Extension Workers then reported CBRHAs’ programmatic data to Woreda Health Officers. Supervision was built into the existing public health monitoring system and Health Extension Workers were not paid for their technical support. CBRHAs referred women who were deemed ineligible due to medical contraindications or other reasons, those who wished to use a different method of contraception, and those clients who could not pay to the nearest government health post or health center. These systems for supervision and referrals highlight the importance of a strong public-private partnership. Supervisors from the Women’s Association of Tigray monitored the supply and demand of DMPA and maintained the financial records of the CBRHAs.

#### Monitoring & evaluation

Monthly client records were completed by CBRHAs and submitted to Health Extension Workers for review, with data from November 2011 to October 2012 available for analysis. These records were aggregated at the woreda level by the MCH Supervisors. Data collection followed a systematic flow of information, which was integrated into the current data collection system of the Tigray Health Bureau, to promote sustainability, accountability and public-private partnership. Monthly programmatic data was collected on the number of new clients served and total injections provided per CBRHA. Monitoring data from the Women’s Association of Tigray Supervisors captured the number of injections provided by CBRHAs for free or below cost, which informed researchers about the level of external support or subsidy likely to be required to sustain the drug revolving fund. The programmatic data allowed researchers to test the financing mechanism of the intervention. Ultimately, these data provided critical information on the feasibility and sustainability of scaling up access to DMPA at the community level through use of CBRHAs in other regions.

## Results

### Characteristics of the Population

The sample (N = 1490) was representative of all women of reproductive age, 15 to 49 years, in the Central Zone of Tigray, Ethiopia (Table 1). The majority (73%) of women were married and over half (54%) had no education. Thirty percent of women were currently using contraception and of those, 69% were using DMPA. Sixteen percent of married women reported an unmet need for contraception and among women who were not currently using contraception, 67% thought they would use a contraceptive method to delay or avoid pregnancy at some time in the future (data not shown). Over 55% percent of women surveyed prefer DMPA as a method of family planning. Yet of the women who prefer DMPA, 63% were not currently using this method (data not shown). Among women who had ever-used or were interested in using DMPA, 68% of respondents said they were willing to pay, and of those willing to pay, 52% were willing to pay 5 birr, which is the current cost of one injection in the project (data not shown).

**Table 1:** Sociodemographic factors among women surveyed (N = 1490).

	%
Age	
15–19	19.3
20–24	17.1
25–29	17.9
30–34	15.7
35–39	13.4
40–44	8.3
45–49	7.5
Marital status	
Never married	13.6
Married/cohabiting	72.3
Divorced/widowed	13.9
Education	
No education	53.6
1–4 years	13.2
5–9 years	22.4
Secondary or greater	10.6
Has had sexual intercourse	85.6
Currently using any contraceptive methods	30.1
Currently using modern contraception	29.5
Currently using injectable contraceptives	20.6
Prefer injectable contraceptives	55.3

### Provider Profile

Table 2 provides a preliminary profile for CBRHAs who completed the training (N = 137). Seventy-four percent of the CBRHAs had previously participated in some level of family planning training by the government or other non-governmental organizations that included commodity distribution, but fewer than half (44%) were providing family planning (pills and condoms) at the time of this project (Table 2). In the past, some CBRHAs were only trained to create awareness about the importance of family planning but were never trained to distribute contraceptive methods. Table 2, shows that while only 11% of CBRHAs reported providing DMPA in the past, 96% believed providing DMPA would improve their services. The vast majority of CBRHAs were comfortable providing DMPA to adolescents and unmarried women, with 98% and 87%, respectively, responding positively (Table 2).

**Table 2:** Background characteristics of Community-Based Reproductive Health Agents who completed training by December 2011 (N = 136*).

	%
Education	
No education	9.0
1–4 years	19.1
5–9 years	58.0
Secondary or greater	13.0
Currently provides family planning	44.4
Average length of time have been providing family planning (years)	1.3
Ever received family planning training	74.2
Ever injected DMPA before training	10.5
Ever injected DMPA before training among those who currently provide family planning	15.3
Thinks providing DMPA will improve services	95.5
Comfortable providing DMPA to adolescents	97.7
Comfortable providing DMPA to unmarried women	86.5

* Missing data for one CBRHA.

### Service Provision

Between October and December 2011, 137 CBRHAs successfully completed training and began delivering services in three woredas of Central Zone. At the time of data collection in October 2012, 134 CBRHAs were still involved in the project. From November 2011 to October 2012, CBRHAs had provided 2541 injections of DMPA. Of these injections, 1185 (47%) were provided to new clients (i.e. either new to DMPA, or new to family planning all together). The average number of injections provided per CBRHA was 19.

### Drug Revolving Fund

DKT provided an initial donation of 2,500 doses of DMPA for the project. An additional 16,000 injections were purchased from DKT by the Women’s Association of Tigray with project funds and distributed to the Women’s Association of Tigray offices in each woreda. CBRHAs provided injections at a cost of 5 birr ($0.29 USD) to women and return to the Women’s Association of Tigray to purchase additional stock. It is still too early to evaluate how much financial support the drug-revolving fund will require and what level of support is sustainable. Implementation in three woredas does not provide enough data to effectively asses the survival of the drug revolving fund based on the initial capital invested. However, from early estimates, it appears that just over 31% of injections have been given for free to the poorest women, including adolescents.

### Rural Social Marketing

The Women’s Association of Tigray, a civil society organization, has played an integral role in raising community awareness of the project through their women’s networks. Each kebele (village cluster) has a women’s network that meets biweekly. The CBRHAs introduced the project and their new role of providing injectable contraceptives during these meetings. It is through these meetings that women in the community heard about other women receiving injections from CBRHAs, which was critical to building trust and confidence. These meetings served as an important venue for rural social marketing.

Also as part of the social marketing campaign, each CBRHA received marketing materials and was involved in the IEC activities in their villages. Together with woreda health officials, CBRHAs sensitized community stakeholders about the new project. The IEC campaign was more successful in some districts than others, which contributed to the woreda-level differences in project implementation. The level of participation of the woreda health officials was indicative of how they valued the program and how they viewed their role within the program, demonstrating that a strong community and stakeholder sensitization program must precede the deployment of CBRHAs in DMPA provision.

## Discussion

Preliminary results from this project demonstrate that the methodology of merging community-based distribution with social marketing offers an opportunity for expanding community access to DMPA with the potential for partial cost recovery and long-term sustainability. Through baseline data collection, we found that many women in the Central Zone of Tigray have unmet need for contraception and this problem is even greater among women who prefer DMPA. Clearly this model of DMPA service delivery is necessary and promising: 47% of clients reached with this intervention were new adopters of this method. The early success of this project is largely a result of providing women with their preferred method. Our findings contrast with recent program experience in Ethiopia, where researchers suspected that their intervention, involving access to credit and/or community-based distribution of condoms and pills in three communities, failed to significantly increase contraceptive uptake compared to a control community because the community health workers could not offer the preferred method, injectable contraceptives [46]. In fact, the authors of that study concluded that CBRHAs should be trained to provide injectable contraceptives in Ethiopia.

The model employed in this study goes beyond method preference and access. Genna et al. [45] investigated the sustainability of community-based distribution programs utilizing CBRHAs to increase contraceptive uptake and found that prevalence fell after the community-based projects ended. Therefore, the sustainability of community-based distribution programs is as important as their effectiveness. With limited funding for reproductive health services in Ethiopia, financial sustainability is a high priority.

Our model was developed with a broader understanding of private sector strategies of demand generation, cost recovery, and willingness to pay. The preliminary results from the drug revolving fund indicate that the model will need external support and subsidization to accommodate women who are not able to pay for services (approximately 31%). While we still need more cost data to fully evaluate the drug-revolving fund, data from year 1 provides insight on the level of support the fund will require for long-term sustainability. Though drug subsidization will be necessary, this model does provide an element of cost recovery. It is important to note that the DKT-procured DMPA cost of 3 birr is already subsidized. Our CBRHAs’ clients are paying 5 birr as printed on the socially marketed DMPA.

Under this model, community-based distribution workers are incentivized to increase sales and programs are potentially less undermined by attrition. Similar efforts have been successfully piloted and scaled up in Madagascar [26], [27]. The community health workers involved in the pilot project in Madagascar are part of the public health system, whereas our model of distribution bypasses the public health system for DMPA procurement and service delivery, with CBRHAs acting as social marketers of DMPA. However, as in Madagascar, under our approach, the public health system is directly involved in supervision and monitoring. By collaborating with Woreda Health Experts and Health Extension Workers, the project benefits from their technical and clinical contributions to supervision and support of widely-dispersed CBRHAs. This private public partnership is extremely important to the feasibility of our model. Monthly supervision of CBRHAs fits into the existing supervision structures of the woreda health offices without overburdening staff, which is especially important to the sustainability of the project.

The desire for confidentiality when using contraception is still extremely high in many villages. This is consistent with other studies in Ethiopia which found the potential for secrecy contributed to the preference for injectable contraceptives [48]. It is clear that the CBRHAs’ services are filling a void and addressing some serious barriers to accessing contraception. However, the private nature of the family planning distribution poses significant challenges to the overt marketing campaign typically employed in urban social marketing of contraceptives. Through monitoring visits, it became clear that the majority of marketing is happening between the clients themselves. In fact, some clients are not interested in public marketing of DMPA, as this will potentially alert their husbands to its availability, who may then deny them access to contraception. The interpersonal communication and marketing of DMPA among clients demonstrated by our preliminary findings mirrors dynamics found in a network analysis of interactions with community health workers’ distribution of contraceptives in Madagascar [41]. At this time, we see no need to change the current mode of informal rural social marketing among community women, since it is clearly working and does not conflict with women’s need for privacy.

### Conclusions

Insights gained from the first year of the model provide a framework for further expansion in Tigray, Ethiopia over the next two years. By building upon the success and learning from the challenges of the intervention, we expect that subsequent expansion will yield improved results. Our experience highlights how program planners can tailor interventions to match family planning preferences, meet demand and create more sustainable contraceptive service provision with greater impact in rural settings.
